# Bacterial Interactions with *Aspergillus fumigatus* in the Immunocompromised Lung

**DOI:** 10.3390/microorganisms9020435

**Published:** 2021-02-19

**Authors:** Anatte Margalit, James C. Carolan, Kevin Kavanagh

**Affiliations:** Department of Biology, Maynooth University, Manooth W23VP22, Kildare, Ireland; anatte.margalit@mu.ie (A.M.); james.carolan@mu.ie (J.C.C.)

**Keywords:** allergy, *Aspergillus*, cystic fibrosis, *Pseudomonas*, polymicrobial, pulmonary

## Abstract

The immunocompromised airways are susceptible to infections caused by a range of pathogens which increases the opportunity for polymicrobial interactions to occur. *Pseudomonas aeruginosa* and *Staphylococcus aureus* are the predominant causes of pulmonary infection for individuals with respiratory disorders such as cystic fibrosis (CF). The spore-forming fungus *Aspergillus fumigatus*, is most frequently isolated with *P. aeruginosa*, and co-infection results in poor outcomes for patients. It is therefore clinically important to understand how these pathogens interact with each other and how such interactions may contribute to disease progression so that appropriate therapeutic strategies may be developed. Despite its persistence in the airways throughout the life of a patient, *A. fumigatus* rarely becomes the dominant pathogen. In vitro interaction studies have revealed remarkable insights into the molecular mechanisms that drive agonistic and antagonistic interactions that occur between *A. fumigatus* and pulmonary bacterial pathogens such as *P. aeruginosa*. Crucially, these studies demonstrate that although bacteria may predominate in a competitive environment, *A. fumigatus* has the capacity to persist and contribute to disease.

## 1. Introduction

By definition, polymicrobial communities are a collection of microbial species that co-exist in a particular habitat [1]. Arising from co-habitation, interspecies interactions occur, and these interactions shape the landscape of the environment in which the microbial communities reside [2]. When that environment exists in humans, such interactions can influence the health status of an individual. In the context of infectious disease, polymicrobial interactions can be synergistic, whereby the combined effect of multiple microbial species is worse than that where individual species act alone [2]. On the other hand, antagonistic interactions arise due to competition, and occur when one species suppresses the other [3]. The mechanisms employed by microbes as a consequence of these interactions is often detrimental to host health. Inter-species interactions may influence microbial pathogenesis by altering microbial virulence factors and disease progression [1]. Thus, an understanding of how polymicrobial communities, interact with each other and with the host is important when deciding appropriate therapeutic strategies [1,4].

Individuals with chronic pulmonary disease such as cystic fibrosis (CF) or chronic obstructive pulmonary disease (COPD) are susceptible to respiratory infections caused by a multitude of microbial species; the filamentous fungus *Aspergillus fumigatus* is the dominant cause of fungal infections in the immunocompromised airways, while the bacterium *Pseudomonas aeruginosa* is the chief cause of bacterial infections [5,6]. Importantly, these pathogens do not act in isolation, rather their development in the airways is governed by their environment and mutual interactions. Co-infection of the lungs by these pathogens is associated with poor prognostic outcomes for the patient, thus, understanding how these interactions affect disease progression can be key to identifying enhanced control mechanisms.

By their very nature, polymicrobial interactions are difficult to dissect and the results are largely dependent upon the in vitro model systems used to analyze these interactions. Nonetheless, the findings arising from such studies increase our understanding of these relationships and may provide a pathway to design new therapeutic targets. This review will highlight some of the studies concerning the interactions that occur between *A. fumigatus* and other common pulmonary pathogens, with a focus on *P. aeruginosa*, and will consider how the findings may influence the pathogenesis of pulmonary diseases caused by the co-existence of these pathogens in the airways.

## 2. Aspergillus fumigatus

*A. fumigatus* is an opportunistic fungal pathogen and the most pathogenic member of its genus [7]. Although its natural ecological niche is the soil, *A. fumigatus* is ubiquitous, existing indoors and outdoors [8]. Because of this, inhalation of conidia is a daily occurrence. *A. fumigatus* is a versatile microorganism that is equipped to survive and propagate in a variety of environments [9]. The fungus possesses a number of features that make it an excellent human pathogen, including the ability to grow at high temperatures and varying pH. *A. fumigatus* can sustain growth above 42 °C, which in the context of human infection is beneficial for maintaining infection during a febrile state [10]. Additionally, it can adapt to the changing pH of the mammalian host by activating a set of pH-responsive genes regulated by the PacC transcription factor [11,12].

Like many pathogens, *A. fumigatus* can form a biofilm which enables persistence in the host. *A. fumigatus* biofilms are formed when conidia and hyphae become embedded in a self-made hydrophobic extracellular matrix composed of glucans, galactomannans, monosaccharides, hydrophobins, and major antigen proteins [13,14]. The emergence of hyphae within biofilm coincides with the production of secondary metabolites (e.g., gliotoxin, fumagillin), antigenic surface molecules (β(1,3) glucans), and antigens (e.g., aspergillopepsin), which the host responds to by inducing a proinflammatory response. Biofilm formation is dependent upon fungal cell density [15], thus, the ability to reach this density threshold may play a factor in the ability of *A. fumigatus* to establish biofilms in the host. Biofilms enable fungal persistence in the pulmonary cavity by providing protection against cells of the immune system and antifungal drugs. Furthermore, in vitro studies have shown that while hyphae contained in biofilms may be inhibited by competing microbes, they are difficult to kill [16,17,18].

The physical size and hydrophobic nature enable *A. fumigatus* conidia to enter the respiratory tract through inhalation, bypass mucociliary clearance, and reach the alveoli. *A. fumigatus* conidia may evade initial host-cell recognition by masking β(1,3)-glucan residues on the condial cell wall with a thin proteinaceous hydrophobic layer called RodA hydrophobin [19]. As conidia germinate, the RodA layer is shed and β(1,3)-glucan residues are revealed, allowing for recognition by cells of the innate immune system [20]. The shedding of RodA also reveals dihydroxynapthalene (DHN)-melanin, a secondary metabolite found in the conidial cell wall. In the environment, DHN-melanin confers resistance against desiccation and damage from UV radiation, and in the host it plays an important role in virulence by scavenging reactive oxygen species (ROS) and protecting conidia against phagocytosis by host cells [21,22,23].

Where the immune system is compromised, conidia germinate and hyphae may form [20]. This may lead to the manifestation of a disease called aspergillosis, the severity of which is determined by the immune status of the host. There are three forms of aspergillosis; allergic aspergillosis, the most common form of which is known as allergic bronchopulmonary aspergillosis (ABPA) is characterized by the induction of an immune response triggered by the secretion of toxins and allergens from the developing fungus. Saprophytic aspergillosis is characterized by the development of aspergilloma (fungal ball) in chronic lung cavities of the pulmonary tissue, such as those caused by tuberculosis [24]. Invasive aspergillosis (IA) is the most devastating form of aspergillosis and is characterized by the dissemination of fungal hyphae throughout the tissues of the affected area. This occurs in the lungs in more than 90% of cases and is called invasive pulmonary aspergillosis (IPA) [25]. IA targets severely immunocompromised individuals including individuals with neutropenia, organ transplant recipients, and chemotherapy patients [7].

*A. fumigatus* is the causative agent of allergic bronchopulmonary aspergillosis (ABPA). It is estimated that 1–2% of asthma patients and 1–15% of CF patients are affected by ABPA [26]. Clinical manifestations of ABPA include wheezing and bronchospasms and for individuals with CF, decline in lung function may occur [27]. For non-CF patients, ABPA diagnostic criteria include asthma, elevated serum levels of *Aspergillus*-specific IgG antibodies, elevated serum levels of IgE and eosinophilia [28,29]. Several of the diagnostic criteria for ABPA are common manifestations of CF, for example, elevated IgG and IgE anti-*A. fumigatus* antibodies are not uncommon in CF serum due to sensitization to *A. fumigatus* in CF [30]. For this reason diagnosis of ABPA in a CF patient may present certain challenges [29]. Nonetheless, in the context of ABPA diagnosis, *A. fumigatus*-specific IgE levels are recognized as the most useful diagnostic tool [30,31].

ABPA is described as a hypersensitivity lung disease in response to bronchial colonization by *A. fumigatus* [32]. It occurs when conidia deposited in the airways begin to germinate and release metabolites such as gliotoxin, fumagillin, and allergens such as Asp f family of allergens [33,34]. These toxins disturb the epithelial barrier and impede mucociliary clearance [35,36]. An influx of pulmonary macrophages and neutrophils mediate a proinflammatory cytokine cascade that promote a Th2-type adaptive immune response involving the release of IL-4, IL-5, IL-9, and IL-13 [37]. IL-4 induces IgE production, which binds to, and sensitizes granulocytes including basophils and mast cells. IL-5 and IL-9 recruit eosinophils and mast cells to the infection site and IL-13 induces mucus hypersecretion, airway fibrosis, and eotaxin production, thus contributing to the eosinophilic inflammatory response [38,39]. These factors contribute to the chronic inflammation that feature heavily in the CF airways.

In the absence of ABPA, the role of *A. fumigatus* in CF is becoming better understood and more appreciated. Until recently, young children with CF were thought to be less affected by *A. fumigatus* than older patients, with a prevalence rate of 6%–25%, compared with up to 57% in adults [40,41]. Traditional culture methods, such as plate assays, likely underestimate the actual prevalence of *A. fumigatus* among this group of patients [42]. The inclusion of molecular methods (qPCR) as a diagnostic tool for *A. fumigatus* infections provide a more accurate scenario and indicate that *A. fumigatus* is more prevalent in juveniles than previously reported [42]. Recent longitudinal studies have provided evidence to support this and *A. fumigatus* infections in children are now recognized as a major contributing factor in lung function decline in this cohort of patients, affecting up to 68% of patients [42,43,44,45]. This is, in part, associated with aggressive antibiotic therapies targeting bacterial pathogens, which thereby provide fungal pathogens with an opportunity to colonize [44]. *A. fumigatus* infection during early childhood is correlated with structural damage to the lung and decline in lung function, and while co-infection with other pathogens exacerbates disease prognosis the long term effects of early exposure to *A. fumigatus* remain to be explored [43,45,46,47].

## 3. Pseudomonas aeruginosa

*P. aeruginosa* is a Gram-negative, rod-shaped bacterium that is ubiquitous in nature, particularly in aquatic and soil environments. Its ubiquity is due to the ability of *P. aeruginosa* to survive in environmental niches that are intolerable to other microorganisms and its nutritional versatility. The genome of *P. aeruginosa* is large (~6.3 kbp) [48], and approximately 8–10% of these genes are predicted to be regulators of gene expression [49]. This confers *P. aeruginosa* with an incredible capacity to adapt rapidly to environmental changes such as nutritional availability [49]. Additionally, *P. aeruginosa* possess several efflux pumps which can expel toxic compounds, such as antibiotics, from the cell faster than they can accumulate [49,50]. A classic feature of chronic infection caused by *P. aeruginosa* is the increased exopolysaccharide production and the emergence of biofilms. Biofilms confer a layer of protection against phagocytic cells such as neutrophils, and antibiotics. Although neutrophils migrate to biofilms, they become immobilized and surrounded by bacteria that escape from biofilms. Neutrophil degranulation is compromised as a result and oxygen consumption by both neutrophils and the biofilm is increased, thereby reducing oxygen availability on the airways [51].

*P. aeruginosa* biofilm formation is dependent upon quorum sensing (QS), the mechanism by which bacteria communicate [52]. QS occurs in a cell density-dependent manner and is necessary for the biosynthesis of secondary metabolites such as pyocyanin and rhamnolipids, which induce neutrophil apoptosis and necrosis, respectively [53,54]. Biofilms are unable to form in the absence of iron and under iron-limiting conditions, QS regulates iron acquisition systems by inducing the production of siderophores such as pyoverdin [55,56].

The switch from non-mucoid to the over-producing alginate mucoid strain is probably the most pronounced phenotypic change that *P. aeruginosa* adopts as it establishes chronic infection [57]. Alginate plays an important role in the maturation and structural stability of *P. aeruginosa* biofilm and increases bacterial evasion of host immune cells and antibiotics [58]. Several loss-of-function mutations that occur during adaptation in the CF lung are characteristic of the establishment of chronic infection, including loss of motility, repression of type three secretion systems and downregulation of QS regulatory genes, such as lasR [59,60,61].

## 4. The Microbial Environment of the Immunocompromised Airways

The microbial environment of the CF airways is an evolving ecosystem, and from infancy, the lungs of CF patients are subject to colonization by a diverse range of microbial species from various genera including *Streptococcus, Prevotella, Rothia, Veillonella,* and *Actinomyces* [62,63]. The CF airways are characterized by an age-related succession of microbial species; in children under the age of 16, *Staphylococcus aureus*, *Haemophilus influenzae,* and *Stenotrophomonas maltophilia* predominate [64,65]. The reduction of infection with *H. influenzae* and *S. aureus* is strongly correlated with increased colonization by *P. aeruginosa* and *Burkholderia* spp., and a decline in lung function [62,65,66]. It is estimated that chronic infection with *P. aeruginosa* affects up to 80% of adults with CF.

Despite the diverse nature of the microbial community that exists in the CF airways, *P. aeruginosa* is consistently identified as the most common pathogen isolated from the lungs of patients after their first decade of life [64,67,68]. Reflected in this observation, in vitro and in vivo interaction studies involving *P. aeruginosa* demonstrate a greater capacity of *P. aeruginosa*, to outcompete other CF-associated species such as *S. aureus* and *H. influenzae* [69,70]. For this reason, *P. aeruginosa* has become the focus for many studies investigating the role of polymicrobial interactions in the context of CF.

Individuals that live with chronic non-cystic fibrosis-related respiratory diseases such as COPD and bronchiectasis are susceptible to infection by pathogens from multiple taxa including *Pasteurellaceae, Streptococcaceae,* and *Pseudomonadaceae* [71]. Microbial diversity is associated with clinical status and is reduced where acute exacerbations occur [71,72]. Two of the most common pathogens detected from the airways of individuals with bronchiectasis are *P. aeruginosa* and *H. influenzae*, however, due to antagonistic interactions that occur between these pathogens, when one is detected, the other is absent [71,72].

The prevalence of *P. aeruginosa* in adults with COPD is estimated to be between 4–15% and higher for individuals with severe COPD and bronchiectasis as part of the diagnosis [73,74]. In contrast, the frequency of chronic *P. aeruginosa* infection for individuals with bronchiectasis as the primary condition is between 9–31% [75]. Compared to infection by other pathogens, *P. aeruginosa* is associated with disease progression, recurrent pulmonary exacerbations, and poorer clinical outcomes, including a higher rate of mortality in patients with bronchiectasis [76,77].

The immunocompromised airways are susceptible to infection by a range of fungal pathogens and several of these, including *Candida* spp., *Cryptococcus* spp., and *Scedosporium aurantiacum* have been studied in the context of co-infection with *P. aeruginosa* [78,79,80]. In all cases, *P. aeruginosa* inhibits fungal growth and/or biofilm formation. Perhaps one of the most fascinating of the fungal-bacterial relationships associated with pulmonary infections, are those arising from the interactions between *Aspergillus fumigatus* and *P. aeruginosa*. *Aspergillus fumigatus* is the most common fungal pathogen isolated from the CF airways. It is detected from early childhood and is persistent in the airways throughout the life of a CF patient [42,64,66]. Longitudinal studies have shown that colonization with *A. fumigatus* is associated with an increased risk of *P. aeruginosa* colonization in CF, and disease prognosis is poor when both pathogens are present [47,81,82,83]. The prevalence of co-colonization with *P. aeruginosa* and *A. fumigatus* in the CF airways is estimated to be between 3.1 and 15.8%, although this occurrence may be higher [47,81,82].

Co-infection with *P. aeruginosa* and *A. fumigatus* have been detected in severe cases of COPD, and the presence of *P. aeruginosa* in the airways is considered a risk factor for *A. fumigatus* infection [84]. *A. fumigatus* is frequently isolated from the airways of individuals with COPD and bronchiectasis, and infection with *A. fumigatus* is a known risk factor for the onset of bronchiectasis in COPD [85,86,87,88]. ABPA is employed as a diagnostic feature of bronchiectasis and can inform treatment programs [89].

Due to the frequency at which these pathogens co-exist in the airways, the interactions that occur between *A. fumigatus* and *P. aeruginosa* are of immense clinical importance in the area of pulmonology. What drives pulmonary exacerbation when the two pathogens are present remains to be fully elucidated [90,91]. The increased number of studies that have begun to investigate *A. fumigatus*–*P. aeruginosa* interactions in the past decade is a reflection on the clinical importance of co-colonization with these pathogens. In general, the results of these studies show that *P. aeruginosa* outcompetes *A. fumigatus*, a finding supported by the predominance of the bacteria in the CF lung.

## 5. The Interactions Between *A. fumigatus* and *P. aeruginosa*

*A. fumigatus* persists in the CF airways throughout childhood and into adulthood, yet despite this, *P. aeruginosa* eventually predominates [42,47,81]. This suggests that interactions with pathogens such as *A. fumigatus* may influence the pathogenicity of *P. aeruginosa* by altering its virulence and the host environment to pave the way for chronic *P. aeruginosa* infection [92].

**Figure 1:**
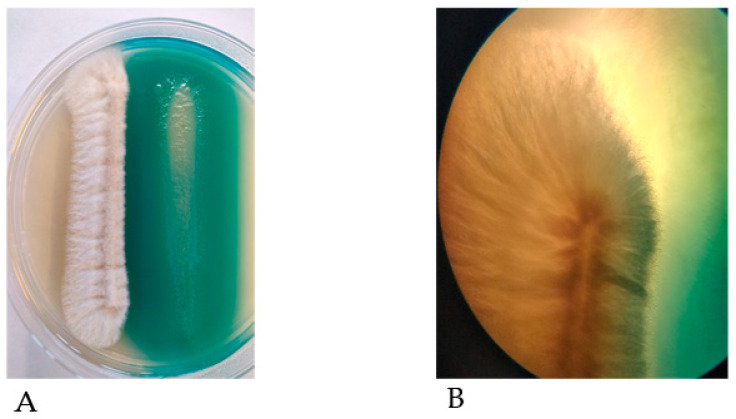
*(***A**) *P. aeruginosa* cells (right) grown alongside *A. fumigatus* conidia (left) on nutrient agar. *P. aeruginosa* inhibits growth of *A. fumigatus* as evidenced by reduced mycelial expansion on the side of bacterial growth. The green pigment produced by *P. aeruginosa* is pyocyanin. (**B**) A magnified image of (A) in which *A. fumigatus* growth is inhibited by *P. aeruginosa*; the expansion of *A. fumigatus* mycelia are inhibited by the close proximity to *P. aeruginosa* cells. By contrast, the absence of bacteria on the left hand side of the fungus allow mycelia to expand outward.

Analysis of the interactions between *A. fumigatus* and *P. aeruginosa* in vitro have revealed several antifungal mechanisms by which *P. aeruginosa* can outcompete *A. fumigatus* [16,93,94,95,96] (Figure 1). *P. aeruginosa* isolates taken from patients with cystic fibrosis have a greater antifungal capacity than non-cystic fibrosis isolates. Non-mucoid isolates are more inhibitory than mucoid isolates, which may explain why *A. fumigatus* is detected at higher levels in older cystic fibrosis patients where chronic (mucoid) *P. aeruginosa* infections are more common [91,97,98,99]. Many of these interaction studies have focused on the direct effects of *P. aeruginosa* on *A. fumigatus*-biofilm formation, on the effects of bacterial biosynthetic products (e.g., phenazines) on the fungal growth and development or, of fungal metabolites on *P. aeruginosa* [95,96,100,101,102].

*P. aeruginosa* secretes a range of compounds that inhibit *A. fumigatus* development and biofilm formation [16,94,95]. Phenazines (pyocyanin, phenazine-1-carboxamide, 1-HP and phenazine-1-carboxylic acid) are QS-regulated redox-active molecules that are important in bacterial respiration and energy production in oxygen-limiting environments such as the CF airways [103]. Phenazines are ROS producing compounds and in the host, changes in the redox balance caused by ROS result in host-cell damage and death [103]. The production of ROS by phenazines also has implications for *A. fumigatus* survival [95]. Phenazines can enter into swollen, but not resting, conidia and target the mitochondria, inducing ROS production [95]. The accumulation of ROS is thought to interfere with *A. fumigatus* growth and biofilms by inducing fungal apoptosis [95,96]. Exposure of *A. fumigatus* biofilms to culture supernatants from non-mucoid and mucoid *P. aeruginosa* CF isolates resulted in a greater increase of ROS in fungal biofilms exposed to the non-mucoid strain [96]. In the CF airways, mucoid strains are associated with the downregulation of QS-regulated molecules including phenazines [104]. This suggests that these antagonistic interactions may occur prior to the switch from non-mucoid to mucoid and the establishment of chronic infection in the CF lung.

The definitive role of phenazines as a fungicidal agent is uncertain, however, as phenazine-deficient mutants have also been shown to inhibit fungal growth, although the authors of this study acknowledge the possible anti-fungal role of an unknown molecule upregulated as a result of phenazine depletion [94]. The *P. aeruginosa* siderophores, pyoverdin and 1-hydroxyphenazine (1-HP), chelate iron in the environment, depriving *A. fumigatus* of a necessary nutrient, thereby suppressing fungal growth and biofilm formation [94,95]. Pyoverdin is thought to be the key component involved in outcompeting *A. fumigatus*, and mutants deficient in pyoverdin biosynthesis were unable to inhibit fungal growth [94].

Another class of *P. aeruginosa* QS-regulated molecules are dirhamnolipids. These biosurfactant molecules alter *A. fumigatus* cell-wall phenotype by interfering with the extracellular matrix, enabling enhanced bacterial binding to the fungus, increasing melanin production and inhibiting β 1,3-glucan synthase, causing the hyphal cell-wall to thicken, thereby suppressing fungal growth development [102]. In co-cultures, *A. fumigatus* stimulates *P. aeruginosa* elastase production, which inhibits the growth of fungus and is also cytotoxic to the alveolar epithelial cells, A549, in vitro [105].

These findings are of clinical relevance because although the arsenal of secondary metabolites secreted by *P. aeruginosa* in the presence of *A. fumigatus* may have anti-fungal properties, these bacterial compounds and the consequences arising from their interactions with *A. fumigatus*, may have negative implications for the host. For example, in vivo, melanin enables fungal evasion of phagocytic activity [106]. *P. aeruginosa* elastases can degrade host antimicrobial surfactant proteins SP-A and SP-D and disrupt tight junctions between epithelial cells [107,108,109]. Phenazines contribute to cytokine-mediated damage to host cells by induce proinflammatory cytokines and siderophores contribute to iron depletion in the host environment [110,111]

Despite the demonstrable ability of *P. aeruginosa* to subdue *A. fumigatus* growth (Figure 1) [16,17,94,95,102], several studies reported the capacity of *A. fumigatus* to compete with *P. aeruginosa* [100,112]. This supports the notion that *A. fumigatus* can persist in the CF airways, despite not being the dominant pathogen. For example, *P. aeruginosa* can inhibit the growth of *A. fumigatus* conidia, but not of preformed hyphae [17]. This may be attributed to the ability of hyphae, but not conidia to produce gliotoxin which has anti-*Pseudomonas* activity [100,113]. *A. fumigatus* produce hydroxamate-containing siderophores (ferricrocin, hydroxyferricrocin, fusarinine C, triacetylfusarinine C) in response to iron limitation. The production of these siderophores can mitigate the effect of *P. aeruginosa* pyoverdin and, in part, protect *A. fumigatus* biofilm, as shown in *A. fumigatus* siderophore-deficient mutants, which are more susceptible to the effects of pyoverdin than the wild-type [112].

*A. fumigatus* secretes a range of degradative enzymes that contribute to the ubiquity of the fungus in nature by supporting fungal growth on plant matter [114,115,116]. Many of these biological determinants also play a role in establishing disease in humans and are associated with virulence and pathogenesis [9,115,116,117,118,119]. How these enzymes directly or indirectly influence bacterial growth has not yet been investigated in detail, however, recent studies have shown that *A. fumigatus* alters the environmental conditions in vitro, by converting a nutrient-poor, nitrate-rich environment into one rich in amino acids. These conditions, known to exist in the CF airways, may enable *P. aeruginosa* to outcompete *A. fumigatus* by promoting a metabolic-driven increase in bacterial growth [113]. Analysis of the culture filtrates produced by *A. fumigatus* identified an abundance of degradative enzymes which are also involved in virulence, including alkaline protease 1, alkaline protease 2, aspergillopepsin-1, and major allergen Asp f 2 [115,117,118,119]. The increase in bacterial growth owing to the presence of *A. fumigatus* may affect the ability of host epithelial cells to efficiently internalize incoming pathogens and participate in microbial clearance [120]. This may be exacerbated by *A. fumigatus*-mediated inhibition of host cell apoptosis [121,122,123].

**Figure 2:**
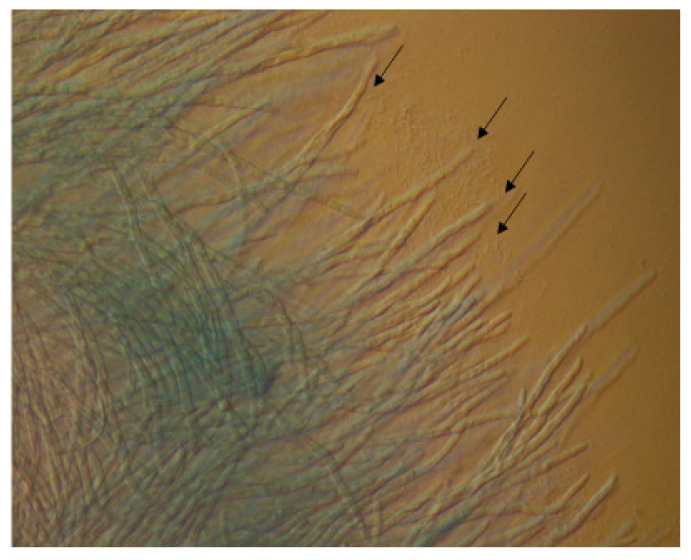
*P. aeruginosa* cells (indicated with black arrows) travel toward *A. fumigatus* mycelia and cluster around the hyphal tips. Viewed through an Olympus BX61 fluorescent microscope (40X).

On semi-solid nutrient agar plates, *P. aeruginosa* cells can travel from one area of the plate toward the developing hyphae of *A. fumigatus* at another area, and form a cluster around the hyphal tips (Figure 2). This may be caused by an area of increased nutrient availability for the bacterial cells and indicates the ability of bacterial cells to interact directly with the fungus.

While the relationship between *P. aeruginosa*–*A. fumigatus* is antagonistic for the most part, there is increasing evidence to show that *P. aeruginosa* volatile organic compounds (VOC) stimulate the growth of *A. fumigatus* without the requirement for direct contact between the pathogens [101,124]. Volatile sulfur compounds (VSC) such as dimethyl sulfide (DMS) released by *P. aeruginosa* provide *A. fumigatus* with a sulfur source, which is necessary for fungal growth [124]. In the CF airways, *P. aeruginosa* releases VOCs [125], thus the VOC-mediated stimulation of fungal growth may facilitate the persistence of *A. fumigatus* in the lungs.

## 6. Interactions Between *A. fumigatus* and Other Pulmonary Pathogens

With the exception of *P. aeruginosa*, the interactions between *A. fumigatus* and other pulmonary pathogens remain relatively unexplored, although this is changing as the recognition for the impact of polymicrobial interactions involving this pathogen on disease progression begin to surface [6]. A better understanding of these dynamics may help predict the treatment regimens necessary to ameliorate pulmonary infections.

While bacteria such as *S. aureus* are associated with chronic colonization in juvenile CF patients, *A. fumigatus* persists throughout the lifetime of individuals with CF, but rarely establishes chronic infection [64,126,127]. Co-cultures of *S. aureus* and *A. fumigatus* conidia revealed antagonistic interactions resulting in the bacteria outcompeting the fungus [18]. In this study, *S. aureus* cells adhered to conidia and fungal-bound bacteria served as a chemoattractant for other bacterial cells. Fungal inhibition by *S. aureus* was most effective where bacteria adhered to the surface first. Bacteria induced lysis of the conidia and interfered with hyphal development [18].

The Gram-positive bacterium, *Streptococcus pneumoniae*, is the causative agent of pneumonia and sepsis in elderly people and children [128,129,130]. These bacteria are also detected in the airways of CF patients and associated with pulmonary exacerbations, particularly in children [131,132,133]. *S. pneumoniae* inhibit the development of *A. fumigatus* in vitro, and disassemble pre-formed fungal biofilm, the mechanism for which is regulated by pneumolysin and hydrogen peroxide, which bacteria produce as a byproduct of aerobic respiration [134].

Although *Klebsiella pneumoniae* is not typically associated with CF infections, it is nonetheless a common cause of pulmonary disease [135,136]. In vitro, in mixed biofilms, *K. pneumoniae* suppressed *A. fumigatus* conidial germination, hyphal development, and biofilm formation without killing the fungus [137]. On the contrary, *K. pneu*m*oniae* biofilm increased in the presence of *A. fumigatus*. These effects were dependent upon direct contact between the fungal and bacterial pathogens in which *K. pneumoniae* induced oxidative stress and upregulation of cell wall synthesis genes in *A. fumigatus* [137].

*Stenotrophomonas maltophilia* is an emerging CF-associated pathogen [138]. Interactions between *S. maltophilia* and *A. fumigatus* were analyzed in mixed biofilms and, similar to that which was observed during studies using *S. aureus* and *K. pneumoniae*, the results showed that *S. maltophilia* interacted directly with fungal biofilm and in the presence of bacteria, *A. fumigatus* hyphal formation was delayed, conidiation was abrogated and biofilm formation was reduced. Moreover, the conidial cell wall was thicker in the presence of *S. maltophilia*.

These interaction studies indicate that while bacteria outcompete *A. fumigatus* in terms of growth, such encounters do not kill the fungus, but rather subdue its ability to become invasive. In the context of CF and asthma, this may be clinically relevant, because although *A. fumigatus* does not become invasive, it does persist and induce prolonged inflammation [139,140]. The propensity for the bacteria discussed here to disrupt fungal biofilm formation suggest that the pathogens do not co-exist in biofilms. However, these bacteria are frequently isolated with *A. fumigatus* from the immunocompromised airways, thereby indicating that although co-infections exist, the pathogens may be spatially segregated. The implications for this is the occurrence of colonization by multiple microbial species in different areas of the respiratory system, which may necessitate tailored therapeutic strategies.

## 7. Conclusions

For a long time, bacteria were thought to be the main drivers of disease in the immunocompromised airways [90]. However, advanced molecular techniques have identified fungal pathogens as a major contributing factor in the onset and development of pulmonary infections. In particular, *A. fumigatus* is recognized as one of the most prevalent pathogens associated with the lungs, rarely becoming invasive, but regularly inducing a hypersensitive response in the patient. The way in which *A. fumigatus* interacts with other members of the pulmonary ecosystem is fundamental to understanding how this pathogen competes with others to establish infection or facilitates the establishment of other pathogens. The consequences of synergistic and antagonistic interactions arising from co-infection with *A. fumigatus* and other bacteria may have serious implications for the respiratory health of patients and the importance of understanding how these pathogens interact is underpinned by the negative impact of co-infection between *A. fumigatus* and *P. aeruginosa* in CF patients. Thus, understanding the dynamics of the relationship between these pathogens is fundamental for the development of targeted therapeutics that may disturb these interactions and improve patient health.

## Data Availability

Not applicable.
